# Road use pattern and risk factors for non-fatal road traffic injuries among children in urban India

**DOI:** 10.1016/j.injury.2009.10.048

**Published:** 2011-01

**Authors:** Rakhi Dandona, G. Anil Kumar, Shanthi Ameratunga, Lalit Dandona

**Affiliations:** a Public Health Foundation of India, New Delhi, India; b George Institute for International Health-India, Hyderabad, India; c Health Studies Area, Administrative Staff College of India, Hyderabad, India; d School of Public Health and George Institute for International Health, University of Sydney, Sydney, Australia; e School of Population Health, University of Auckland, Auckland, New Zealand; f Institute for Health Metrics and Evaluation, University of Washington, Seattle, USA

**Keywords:** Children, India, Pedestrian, Risk factors, Road traffic injuries

## Abstract

**Objective:**

We assessed the road use pattern and incidence and risk factors of non-fatal road traffic injuries (RTI) among children aged 5–14 years in Hyderabad, India.

**Methods:**

In a cross-sectional population-based survey, data were collected on 2809 participants aged 5–14 years (98.4% participation) selected using multi-stage cluster sampling. Participants recalled non-fatal RTI during the previous 3 and 12 months. RTI was defined as an injury resulting from a road traffic crash irrespective of severity and outcome.

**Results:**

Boys (11.5) had a higher mean number of road trips per day than girls (9.6), and the latter were more likely to walk and less likely to use a cycle (*p* < 0.001). With increasing household income quartile, the proportion of trips using cycles or motorised two-wheeled vehicles increased while trips as pedestrians decreased (*p* < 0.001). Based on the 3-month recall period, the age-sex-adjusted annual rate of RTI requiring recovery period of >7 days was 5.8% (95% CI 4.9–6.6). Boys and girls had similar RTI rates as pedestrians but boys had a three times higher rate as cyclists. Considering the most recent RTI in the last 12 months, children of the highest household income quartile were significantly less likely to sustain pedestrian RTI (0.26, 95% CI 0.08–0.86). The odds of overall RTI were significantly higher for those who rode a cycle (2.45, 95% CI 1.75–3.42) and who currently drove a motorised two-wheeled vehicle (2.83, 95% CI 1.60–5.00).

**Conclusion:**

These findings can assist in planning appropriate road safety initiatives to reduce cycle and pedestrian RTI among children to reduce RTI burden in India.

## Introduction

Unintentional injuries are important contributors to the preventable causes of mortality and morbidity among children worldwide.^35^ The vast majority of child injury deaths occur in low- and middle-income countries where the injury mortality rate among those aged less than 15 years is five times higher than that in the high-income countries.^35,49^ Drowning and road traffic injuries (RTI) are the two largest causes of injury mortality among children aged 5–14 years.^26,28,35,48^

The burden of disease due to injuries is increasing in the developing countries.^10,27,35^ RTI is the second leading cause of death among those 5–14 years of age worldwide with an estimated 180,000 children 14 years of age or less killed every year.^36^ While India still continues to address infectious conditions among children, injuries are emerging as a considerable public health problem with India having one of the highest childhood disability-adjusted life year rates attributable to injuries.^6,7,10,16^ RTI were the second leading cause of death and burden of disease among 5–14 years old in India in the year 2000.^26^

We assessed the road use pattern, incidence and risk factors for non-fatal RTI among those aged 5–14 years in urban India. Such data are an important input into decision-making related to designing of appropriate RTI policies and intervention programmes and for adapting the existing ones from developed countries to reduce the RTI burden in this age group in India.

## Materials and methods

This study was approved by the Ethics Committee of the Administrative Staff College of India, Hyderabad, India, and the research conformed to the principles embodied in the Declaration of Helsinki. The detailed methodology of this study on people aged 5–49 years is published elsewhere,^6,7^ and the details relevant to this paper are presented.

### Study design

This study was conducted from October 2005 to December 2006 in Hyderabad city (population of 3.8 million in 2001).^40^ Hyderabad city was divided into 2542 clusters of mostly 1400–1600 population and listed in sequence of which 50 clusters were selected using a three-stage systematic cluster sampling procedure with equal probability of selection based on their socioeconomic profile. We also selected one cluster of 49 homeless persons to represent this group in the population. Within each cluster (other than the homeless cluster), we enumerated the households and residents in each household. A household was defined as persons eating from the same kitchen. Hostels, hotels, commercial establishments, and prisons were not included. All residents 5–49 years of age in the selected clusters were considered eligible. Systematic sampling, with the first number drawn randomly, was carried out to sample households with the aim of sampling 215–225 eligible people in each cluster. This usually required a sampling interval between 5 and 8 households depending on the total number of households and the eligible population in a cluster. All members aged 5–49 years in the selected households were sampled. Assuming 85% participation rate, a sample of 11,097 people aged 5–49 years was recruited for the survey.

### Interviews

Trained interviewers obtained written informed consent from eligible people for participation in the study, followed by a confidential interview using a questionnaire designed for this study. Detailed demographic data were obtained for all participants aged 5–49 years. Information was collected from parent/guardian for participants less than 11 years of age, and in the presence of parent/guardian for those aged 11–14 years.

Of relevance to this paper, the participants were asked details of their road use including the average number of trips and time spent on road per day, mode of transport, and whether they could ride a cycle or drive a motorised two-wheeled vehicle. The journey from the point of origin to the destination was considered as a trip, and was explained by giving appropriate examples (such as home to school, home to market, play area to home), and they were asked about average number of trips that they made on road and the time spent in these trips on a usual day. The participants recalled if they were involved in road traffic crash (RTC) in the preceding 3 and 12 months. They were then asked about any injury resulting from RTC irrespective of the severity. Detailed data including duration of RTI, vehicles involved, days of recovery/disability, and leaves taken from school/work were documented. If a participant reported more than one RTI during the recall periods, all RTI were documented.

RTI was defined as any injury resulting from RTC irrespective of severity and outcome. RTC was explained to the respondent as any crash on a road involving at least one moving vehicle irrespective of it resulting in an injury. This could include collision with a vehicle or any non-moving object while driving/riding a vehicle, skidding/slipping/overturning of a moving vehicle while driving/riding a vehicle, collision with a moving vehicle while walking/running/standing/sitting on road, or fall from a moving vehicle. These explanations were given to each participant before asking questions on RTC and RTI.

### Data management and analysis

Data were entered in an MS Access database and data entered by one data entry operator were checked by another. SPSS was used for statistical analysis. Road use pattern is reported for boys and girls. Annual non-fatal RTI rates are calculated using 3-month recall periods for overall non-fatal RTI and for RTI as recall bias is a major limitation for data on non-fatal injuries, and less severe injuries in particular are underestimated with longer recall periods.^6,29,33^ We calculated the annual rates for RTI requiring recovery period of ≤7, 7–29 and >29 days for boys and girls wherein the recovery period was defined as days taken to return to normal daily activities as prior to RTI. The RTI rates were adjusted for the age distribution of Hyderabad population, and the 95% confidence interval (CI) include the design effect (DE) for the cluster sampling strategy.^2^ The incidence rates are not adjusted for exposure. The incidence of non-fatal RTI as a pedestrian, cycle user, and motorised two-wheeled vehicle user are presented.

The characteristics of the crash resulting in RTI are presented for the most recent non-fatal RTI in the last 12 months. Univariate and multivariate analyses were performed to understand the association of a variety of risk factors for non-fatal RTI-overall, as a pedestrian and as a cycle user. In the multiple logistic regression models, the effect of each category of a multi-categorical variable was assessed by keeping the first or the last category as reference, and all the variables were introduced simultaneously in the models. Chi-square test for significance is reported where appropriate. Per capita monthly income of household was considered in four quartiles based on the distribution in the study population.

Details of the injuries sustained, treatment sought, recovery period, and leave taken are presented. For participants who were still recovering at the time of interview and those who had not recovered, recovery period was taken to be the length of time since sustaining RTI. Estimates for the recovery days and school-person-days lost in Hyderabad annually are presented.

## Results

A total of 2809 (98.4%) of the 2856 eligible participants aged 5–14 years participated in the study. 1425 (50.7%) were aged 5–9 years and 1460 (52%) were boys. Two hundred and fourteen children (7.6%) were currently employed for work (Table 1).

### Road use pattern

Table 1 summarises the road use details. The average number of trips on road per day was significantly higher for boys (mean 11.5, median 10) than for girls (mean 9.6, median 8). Girls were significantly more likely to walk and less likely to use a cycle for trips in a day when compared to boys (*p* < 0.001). The overall mean time spent on the road per day by children was 2.14 h (median 2.0), and boys spent significantly more time on the road (mean 2.35 h, median 2.16) than girls (mean 1.93, median 1.66) (*p* < 0.001).

Among the 1045 (37.2%) children who knew how to ride a cycle, 291 (27.8%) were 5–9 years of age. All of the 85 children who currently drove a motorised two-wheeled vehicle were aged 12–14 years (mean 13.6 years), none had a driving license, none reported wearing a helmet, and the mean age at which they started driving a motorised two-wheeled vehicle on the road was 12.5 years. They were significantly more likely to be employed as compared with being a student (*p* = 0.007).

**Fig. 1:**
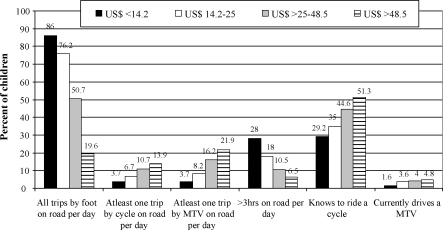
Road use pattern among children aged 5–14 years based on the per capita monthly household income quartiles. Data on per capita monthly income were not available for 90 children. Trips by cycle and motorised two-wheeled vehicles (MTV) include trips as pillion riders.

Significant differences were found in the road use pattern for the children based on the per capita monthly household income (Fig. 1). All trips by foot and the time spent on road per day decreased with increasing per capita household income quartile (*p* < .0.001). On the other hand, the proportion of trips by cycle (*p* < 0.001) or motorised two-wheeled vehicle (*p* < 0.001), knowing how to ride a cycle (*p* < 0.001) or currently driving a motorised two-wheeled vehicle (*p* = 0.002) increased with increasing per capita monthly household income quartile.

### Incidence of non-fatal RTI

**Fig. 2:**
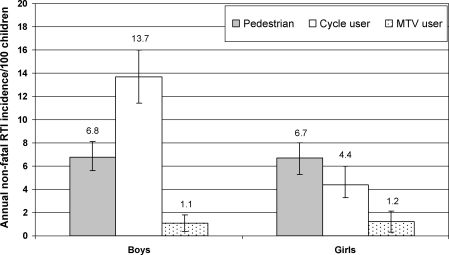
Annual incidence of non-fatal road traffic injury (RTI) using 3-month recall period as a pedestrian, cycle user and user of motorised two-wheeled vehicle (MTV) among boys and girls aged 5–14 years. The bars denote 95% confidence interval.

Based on the 3-month recall period, the annual age-sex-adjusted rate for overall RTI was 18.5% (95% CI 16.8–20.3; DE 1.5) and for RTI requiring recovery period of >7 days was 5.8% (95% CI 4.9–6.6; DE 1.05). The age-adjusted RTI rates for boys were 23.6% (95% CI 20.6–26.6; DE 1.83), 15.2% (95% CI 12.6–17.7; DE 1.91), 6.1% (95% CI 4.8–7.3; DE 1.04) and 0.9% (95% CI 0.3–1.5; DE 1.51) for overall RTI and RTI requiring recovery period of ≤7, >7–29, and >29 days, respectively. These rates for girls were 13.1% (95% CI 11.1–15.0; DE 1.19), 8.1% (95% CI 6.5–9.6; DE 1.14), 3.6% (95% CI 2.6–4.5; DE 0.94), and 0.9% (95% CI 0.6–1.2; DE 0.42), respectively. Boys and girls had similar RTI rates as a pedestrian and motorised two-wheeled vehicle user, but boys had three times higher incidence of RTI as a cycle user as compared with girls (Fig. 2).

### Characteristics of non-fatal RTI

Based on 12-month recall period, 263 episodes of RTI were reported for 237 children (11% reported more than one episode). Table 2 summarises the characteristics of the 237 most recent non-fatal RTI episodes of which 109 (46%) were as a cycle user, 101 (42.6%) as a pedestrian, 20 (8.4%) as motorised two-wheeled vehicle user and 7 (3%) as other vehicle occupant. A major proportion of the crashes resulting in RTI occurred between 1500 and 1800 h (44.3%) and while on a trip not from/to school or work (81%). Nearly 95% of RTI as a cycle user and 15% of RTI as a motorised two-wheeled vehicle user was as a driver, and 24.8% of RTI as a pedestrian occurred while playing on the road.

Collision with another vehicle was the cause of crash in 61.6% of the crashes and vehicle skidding was responsible for 22.3% of the crashes. Presence of sand and small stones on the road accounted for the majority of road-related reasons for vehicle skidding. Sudden application of brakes (due to appearance of a vehicle/person/animal) resulting in fall due to loss of balance were responsible for the majority of vehicle skidding/fall which was not due to road-related reasons.

### Risk factors for non-fatal RTI

On applying multiple logistic regression to the 237 most recent RTI in the last 12 months (Table 3), children who had >15 trips per day on the road had four and two times the odds of RTI as a pedestrian and overall RTI, respectively. Those who rode a cycle and currently drove a motorised two-wheeled vehicle had significantly higher odds of overall RTI. Boys and those who rode a cycle were significantly more likely to have RTI as a cycle user, and those belonging to income quartile IV were significantly less likely to have RTI as a pedestrian. Mother's education level was significantly associated with overall RTI and RTI as a pedestrian. No significant interaction was found between the average number of trips and hours spent on road per day in the logistic models.

### Injuries, recovery and time off work or school

Among the 237 cases of the most recent RTI in the last 12 months, the majority reported injuries on legs (84%) and hand/arm (61.2%), followed by face (16.5%) and head (4.6%). Two (0.8%) reported having not recovered fully following the injuries and 6 (2.5%) were still recovering at the time of interview. A total of 136 (57.4%) had sought medical treatment for RTI as an out-patient and 2 (0.8%) as in-patient.

**Fig. 3:**
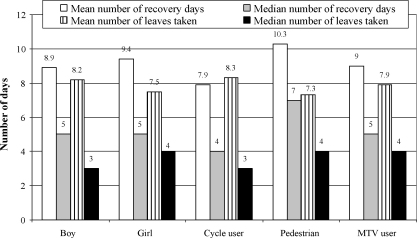
Recovery days and leaves taken for to the most recent road traffic injury (RTI) in the last 12 months for the two sexes and types of road user among children aged 5–14 years. MTV denotes motorised two-wheeled vehicle.

Fig. 3 shows the recovery days and leave days taken due to the most recent RTI for the two sexes and type of road user. The mean number of recovery days required was 9.1 days (median 5.0; range 1–126 days), and RTI as a pedestrian required relatively more recovery days as compared with RTI as a cycle or motorised two-wheeled vehicle user (Fig. 3). Of the 213 children with the most recent non-fatal RTI who were currently in school, 113 (53.1%) reported taking leave from school for that RTI. Of the 24 children with RTI who were employed, 5 (20.8%) took leave from work. The mean number of leave days taken from school/work was 7.94 (median 3.0, range 1–120 days). Two children (1.7%) lost their schooling year due to RTI.

## Discussion

This population-based study describes the road use pattern, and context and risk factors for RTI in children from a large city in India. Extrapolating from a 3-month recall period, we found that 5.8% of children aged 5–14 years had experienced a non-fatal RTI during the previous year which had required a recovery period of more than 7 days. The majority of these RTI were sustained by cyclists or pedestrians. These data confirm the need to consider injuries experienced by children, particularly those who are vulnerable road users, as a public health priority in urban India.

To the best of our knowledge, these are the first population-based data on road use pattern of children from a developing country setting. A distinct difference in the road use pattern was seen between boys and girls and based on socioeconomic strata. Girls were less likely to use a cycle and were more likely to walk. This is probably because boys are given more freedom and are less restrained by the parents to move around as compared with girls.^3,42^ This is also reflected in the larger number and variety of cycles available for boys than for girls in India.^19–21^ It is conceivable that the households with lower income are less able to afford a cycle for their children and hence the children belonging to the lower per capita household income quartile were the least likely to be making road trips using a cycle.

A high number of road trips per day were reported for children with the majority making six or more road trips. It is unlikely that the trips were over-reported as the interviewers asked the respondent to mention each road trip on usual days which were then counted to arrive at the number of trips. A little over 80% of RTI were reported during trips which were not related to school/work. More information including qualitative research that explores the reasons and context for these trips is needed to identify the most appropriate road safety interventions that can reduce RTIs in this age group.

Though only 3% of children were reported to be currently driving a motorised two-wheeled vehicle, they were significantly more likely to be injured in RTC. This finding is of concern as they were driving a vehicle at an age below the legal age of obtaining a learners license for a motorised two-wheeled vehicle with 50cc engine or less in India (16 years).^47^ In addition, none of them reported using a helmet which increases their vulnerability for head injury in case of a crash. We have previously reported that 7.3% of motorised two-wheeled vehicle drivers currently aged 16 years or more in Hyderabad had started driving the motorised two-wheeled vehicle below the age of 16 years.^8^ Stricter law enforcement is needed to prevent such driving by children,^9,35^ and measures involving parents, schools and alternative safe public transport systems can also be explored to reduce underage driving on roads.

The annual rate of non-fatal RTI requiring a recovery period of more than 7 days for boys and girls in our study was 7.0% and 4.5%, respectively. Compared with the relatively sparse data on childhood RTI reported from many developing countries,^15,17,22,25,30,32,34^ the RTI rate was higher in our study. We have previously reported that the relatively higher magnitude of RTI in this population may be related to the methods that we used, which included detailed explanation and probing as well as a short recall period of 3 months to estimate the annual rate.^6^ It is important to note that the incidence of minor non-fatal RTI may be underreported in our study because the information on RTI was documented from parents/guardian for children aged 10 years or less, and those 11 years of age may have underreported RTI in the presence of their parents/guardian. The over-representation of cyclists and pedestrians and preponderance of children among RTI victims has been reported in several low- and middle-income countries.^22–24,35,36,39,43,44,46^

Globally, a variety of risk factors relating to the child, vehicle and environment have been identified for unintentional injuries in children, and these the risk factors can vary from one setting to another.^1,5,11–13,18,35,38,41,45^ We found a higher RTI risk as a cycle user for boys and among those who could ride a cycle. One-third of the cycle injuries were a result of collision with another vehicle and nearly 45% due to skidding/fall from cycle. Cycle is primarily used as a mode of transport and not for recreation in India by children and adults and without a helmet. In addition to increased access to cycles for boys, exposure and mixed traffic patterns are among the major risks for RTI as a cycle user.^4,31^ Pedestrian injuries in children are known to be the highest in Asia and Africa.^22,28^ These data also highlight the protective effect of higher per capita monthly household income on pedestrian RTI. Most pedestrian injuries occurred while walking, crossing or playing on road. It is not uncommon for children to play on roads in India as not many neighbourhoods have playgrounds and residential areas often do not have speed or traffic volume restrictions thereby increasing the risk of RTI for children. They are often unsupervised while playing or while running errands to the local market, and research has shown increased RTI risk with the lack of parental supervision,^37^ and that parental supervision can reduce the RTI risk in children.^14^ Physical and cognitive developmental factors also increase the risk of RTC among young pedestrians.^35^

Injuries to arms and legs were the most common types of injuries sustained, and more than half of the injured children reported seeking medical care as an out-patient. Fractures to arms and legs are reported to be the most common injuries requiring hospital admissions for children.^50^ The injury severity and burden are further highlighted by the days needed for recovery and days lost from school and work.

The specific road use pattern and RTI data presented in this paper can be utilised to adapt for our setting the proven strategies used in high-income countries to reduce RTI in children,^35^ and to target appropriate sub-populations for these strategies. Further studies that explore the particular relationships between the transport and social environments and the communities in which children are raised and experience RTI could guide further development of interventions that can respond appropriately and efficiently to the Indian context.

## Conflict of interest

None.

## Figures and Tables

**Table 1:** Road use pattern for boys and girls aged 5–14 years in Hyderabad.

Variable	Boys (1460) *N* (%)^a^	Girls (1349) *N* (%)^a^	Total (2809) *N* (%)^a^
Age group (years)			
5–9	741 (50.8)	684 (50.7)	1425 (50.7)
10–14	719 (49.2)	665 (49.3)	1384 (49.3)

Occupation			
Student	1334 (91.4)	1261 (93.5)	2595 (92.4)
Employed	126 (8.6)	88 (6.5)	214 (7.6)

Average number of trips on road per day†			
≤5	161 (11)	299 (22.2)	460 (16.4)
6–10	636 (43.6)	617 (45.7)	1253 (44.6)
11–15	356 (24.4)	231 (17.1)	587 (20.9)
16–20	202 (13.8)	129 (9.6)	331 (11.8)
>20	105 (7.2)	73 (5.4)	178 (6.3)

All road trips for the day were on foot†	900 (61.7)	926 (68.6)	1826 (65.1)

Average time spent on road per day (h)†			
≤1.30	286 (19.6)	458 (34)	744 (26.5)
>1.30–3.00	849 (58.2)	694 (51.4)	1543 (54.9)
>3.00	325 (22.3)	197 (14.6)	522 (18.6)

Can ride a cycle†	747 (51.2)	298 (22.1)	1045 (37.2)
Currently drives a motorised two-wheeled vehicle†	76 (5.2)	9 (0.7)	85 (3)

a Percent of column total.

† Chi-square test for difference between boys and girls: *p* < 0.001.

**Table 2:** Characteristics of crash that resulted in the most recent non-fatal road traffic injuries (RTI) using 12-month recall period among children aged 5–14 years in Hyderabad.

Variable	Non-fatal RTI as				All RTI (237) *N* (%)^a^
	Cycle user (109) *N* (%)^a^	Pedestrian (101) *N* (%)^a^	Motorised two-wheeled vehicle user (20) *N* (%)^a^	Other vehicle occupant (7) *N* (%)^a^	
Time of crash (h)†					
0600–1100	12 (11)	21 (20.8)	2 (10)	1 (14.3)	36 (15.2)
1101–1500	21 (19.3)	21 (20.8)	6 (30)	1 (14.3)	49 (20.7)
1501–1800	57 (52.3)	41 (40.6)	4 (20)	3 (42.9)	105 (44.3)
1801–1900	13 (11.9)	9 (8.9)	1 (5)	0	23 (9.7)
1901–0559	6 (5.5)	9 (8.9)	7 (35)	2 (28.6)	24 (10.1)

Reason for being on the road‡					
Going/coming from school/work	11 (10.1)	31 (30.7)	1 (5)	2 (28.6)	45 (19)
Going/coming from elsewhere	98 (89.9)	70 (69.3)	19 (95)	5 (71.4)	192 (81)

Activity at the time of crash§					
Driving a vehicle	103 (94.5)	Not applicable	3 (15)	1 (14.3)	107 (45.1)
Riding a vehicle (passenger)	6 (5.5)	Not applicable	17 (85)	6 (85.7)	29 (12.2)
Walking	Not applicable	41 (40.6)	Not applicable	Not applicable	41 (17.3)
Crossing road	Not applicable	31 (30.7)	Not applicable	Not applicable	31 (13.1)
Playing on road	Not applicable	25 (24.8)	Not applicable	Not applicable	25 (10.5)
Other	0	4 (4)	0	0	4 (1.7)

Cause of crash§					
Collision with a vehicle	37 (33.9)	97 (96)	10 (50)	2 (28.6)	146 (61.6)
Vehicle skid/fell due to reasons not related to road	22 (20.2)	Not applicable	2 (10)	0	24 (10.1)
Vehicle skid due to road-related reasons	26 (23.9)	Not applicable	3 (15)	0	29 (12.2)
Other	24 (22)	4 (4)	5 (25)	5 (71.4)	38 (16)

Other party in the crash§					
Cycle	11 (26.8)	30 (29.7)	0	0	41 (17.3)
Motorised two-wheeled vehicle	20 (18.3)	51 (50.5)	5 (25)	1 (14.3)	77 (32.5)
Motorised three-wheeled vehicle^b^	4 (3.7)	17 (16.8)	2 (10)	0	23 (9.7)
None	70 (64.2)	0	9 (45)	5 (71.4)	84 (35.4)
Other	4 (3.7)	3 (3)	4 (20)	1 (14.3)	12 (5.1)

a Percent of column total.

b Motorised three-wheeled vehicle includes commercial passenger vehicles.

† Chi-square test for significance: *p* = 0.005.

‡ Chi-square test for significance: *p* = 0.001.

§ Chi-square test for significance: *p* < 0.001.

**Table 3:** Association of select variables with the risk of non-fatal road traffic injuries (RTI) using 12-month recall period among children aged 5–14 years using multiple logistic regression.

Variable	Total (2809)	Number with overall RTI (% of total)	Odds ratio for non-fatal RTI in the last 12 months (95% confidence interval)		
			Overall	As cycle user	As pedestrian
Age group (years)*					
5–9	1425	99 (6.9)	1.00	1.00	1.00
10–14	1384	138 (10)	1.03 (0.74–1.43)	1.61 (0.989–2.66)	0.82 (0.54–1.26)

Sex†					
Boy	1460	158 (10.8)	1.24 (0.90–1.69)	1.94 (1.16–3.25)	0.93 (0.61–1.40)
Girl	1349	79 (5.9)	1.00	1.00	1.00

Per capita monthly household income quartiles‡					
US$ <14.2	1081	86 (8)	1.00	1.00	1.00
US$ 14.2–25	674	60 (8.9)	1.08 (0.75–1.57)	1.05 (0.60–1.83)	1.07 (0.64–1.79)
US$ >25–48.5	531	57 (10.7)	1.24 (0.81–1.88)	1.37 (0.75–2.51)	1.06 (0.57–1.94)
US$ >48.5	433	31 (7.2)	0.76 (0.42–1.38)	0.97 (0.42–2.22)	0.26 (0.08–0.86)

Currently in school§					
Yes	2595	213 (8.2)	1.00	1.00	1.00
No	214	24 (11.2)	1.20 (0.71–2.04)	0.79 (0.35–1.77)	1.81 (0.88–3.70)

Average number of trips on road per day#					
0–5	460	20 (4.3)	1.00	1.00	1.00
6–15	1840	145 (7.9)	1.34 (0.74–2.42)	1.18 (0.47–2.92)	2.21 (0.79–6.20)
>15	509	72 (14.1)	2.18 (1.07–4.46)	1.99 (0.68–5.85)	4.02 (1.24–13.04)

Average number of hours on road per day**					
≤1.30	744	38 (5.1)	1.00	1.00	1.00
>1.30–3.00	1543	128 (8.3)	1.36 (0.86–2.13)	1.84 (0.92–3.68)	1.12 (0.57–2.22)
>3.00	522	71 (13.6)	1.97 (1.10–3.51)	1.87 (0.77–4.51)	1.67 (0.71–3.91)

Can ride a cycle††					
No	1764	96 (5.4)	1.00	1.00	Not applicable
Yes	1045	141 (13.5)	2.45 (1.75–3.42)	8.80 (4.62–16.75)	

Currently drives a motorised two-wheeled vehicle‡‡					
No	2724	215 (7.9)	1.00	Not applicable	Not applicable
Yes	85	22 (25.9)	2.83 (1.60–5.00)		

Mother's education level§§					
No education	893	69 (7.7)	1.00	1.00	1.00
Class 1–10	1289	123 (9.5)	1.47 (1.03–2.08)	0.95 (0.57–1.58)	2.09 (1.26–3.47)
More than class 10	541	41 (7.6)	1.22 (0.70–2.13)	1.02 (0.48–2.20)	1.66 (0.68–4.08)

Mother working status##					
Not working	702	63 (9)	1.00	1.00	1.00
Working	2014	169 (8.4)	0.98 (0.71–1.36)	0.78 (0.48–1.26)	1.34 (0.85–2.13)

Number of siblings***					
None	175	19 (10.9)	1.00	1.00	1.00
1	884	80 (9)	0.82 (0.47–1.45)	0.67 (0.31–1.45)	1.19 (0.44–3.19)
2 or more	1707	137 (8)	0.62 (0.35–1.09)	0.56 (0.26–1.21)	0.86 (0.33–2.28)

* Chi-square test for significance: *p* = 0.004, <0.001, and <0.001 for overall, cycle user and pedestrian, respectively.

† Chi-square test for significance: *p* < 0.001.

‡ Data not available for 90 children; Chi-square test for significance: *p* = 0.182, 0.236, and 0.001 for overall, cycle user and pedestrian, respectively.

§ Chi-square test for significance: *p* = 0.128, 0.654, and 0.440 for overall, cycle user and pedestrian, respectively.

# Chi-square test for significance: *p* < 0.001, 0.419, and 0.137 for overall, cycle user and pedestrian, respectively.

** Chi-square test for significance: *p* < 0.001 for overall and pedestrian, and 0.003 for cycle user.

†† Chi-square test for significance: *p* < 0.001 for overall and cycle user.

‡‡ Chi-square test for significance: *p* < 0.001 for overall.

§§ Data not available for 86 children; Chi-square test for significance: *p* = 0.218, 0.071, and 0.024 for overall, cycle user and pedestrian, respectively.

## Data not available for 93 children; Chi-square test for significance: *p* = 0.634, 0.597, and 0.252 for overall, cycle user and pedestrian, respectively.

*** Data not available for 43 children; Chi-square test for significance: *p* = 0.354, 0.686, and 0.294 for overall, cycle user and pedestrian, respectively.
